# Postcoital Bleeding: A Review on Etiology, Diagnosis, and Management

**DOI:** 10.1155/2014/192087

**Published:** 2014-06-17

**Authors:** Christopher M. Tarney, Jasmine Han

**Affiliations:** ^1^Department of Obstetrics and Gynecology, Womack Army Medical Center, 2817 Reilly Road, Fort Bragg, NC 28307, USA; ^2^Division of Gynecology-Oncology, Department of Obstetrics and Gynecology, Womack Army Medical Center, 2817 Reilly Road, Fort Bragg, NC 28307, USA

## Abstract

Postcoital bleeding refers to spotting or bleeding that occurs after intercourse and is not related to menstruation. The prevalence of postcoital bleeding ranges from 0.7 to 9.0 percent of menstruating women. There are multiple etiologies for this common complaint in which most are benign such as cervicitis or cervical polyps. However, the most serious cause of postcoital bleeding is cervical cancer. There are currently no recommendations from governing bodies such as the American College of Obstetricians and Gynecologists on evaluating and treating women with postcoital bleeding. The purpose of this paper is to discuss the common causes of postcoital bleeding, the etiologies of postcoital bleeding, and the likelihood that malignancy is the underlying cause. After an extensive literature review, we compiled a paper illustrating the key concepts a practitioner should know when it comes to postcoital bleeding. Finally, this review will conclude with treatment options for women who are found to have an identifiable source for their bleeding and a discussion on the natural history of postcoital bleeding in women who are found to have no identifiable etiology on evaluation.

## 1. Introduction

Vaginal bleeding not related to menstruation is a common multifactorial gynecologic complaint seen by the primary care clinician and is a source of distress both to provider and patient as this can be a sign of underlying malignancy. Postcoital bleeding consists of spotting or bleeding that is not related to menstruation and occurs during or after sexual intercourse. The point prevalence ranges from 0.7 to 9.0% with one report indicating that the annual cumulative incidence is 6% among menstruating women [1–3]. For premenopausal women who are naturally menstruating, spontaneous resolution has been documented in 51% at two years with no further signs of recurrence [4]. About 30% of patients with postcoital bleeding also experience abnormal uterine bleeding and 15% have dyspareunia [5, 6].

Postcoital bleeding mainly comes from surface lesions of the genital tract to include cervical polyps, cervicitis, ectropion, cervical intra-epithelial lesion (CIN), or carcinoma [7]. The prevalence of cervical cancer in women with postcoital bleeding is 3.0 to 5.5% and prevalence of CIN is 6.8% to 17.8% [6, 8–13]. The large range in prevalence is due to variations in study design, but more importantly on study location. Studies performed in developed countries have a lower prevalence of cervical cancer and CIN due to access to screening programs [10–13]. The American College of Obstetricians and Gynecologists and the Society for Gynecologic Oncologists have no recommendations on the evaluation of postcoital bleeding in menstruating women. In the United Kingdom, there are also no established guidelines to ensure consistent practice. The United Kingdom Department of Health reported in The Guidelines for Suspected Cancer that urgent referral (within 2 weeks) should be made for women more than 35 years of age with postcoital bleeding for more than 4 weeks due to elevated risk for underlying cervical cancer and early referral (within 4–6 weeks) may be made in all other cases of unexplained postcoital bleeding [14]. These recommendations are refuted by Khattab et al. who report that there is no significant difference in the prevalence of cervical cancer or CIN in women either older or younger than 35 years [15].

The Royal Australian College of Obstetricians and Gynaecologists, The Royal Australian College of General Practitioners, The Australian Society for Colposcopy and Cervical Pathology, and the Commonwealth Department of Human Services and Health report that colposcopy should be the primary diagnostic procedure in evaluating women with persistent postcoital bleeding and have a suspicious lesion on their cervix or women with a friable cervix [7]. Nevertheless, these governing bodies report that postcoital bleeding alone is not an absolute indication for colposcopy [16].

The purpose of this paper is to discuss different etiologies of postcoital bleeding, to examine the current literature regarding diagnostic evaluation, and to review treatments of this concerning symptom according to underlying etiology. Currently, there is no evidence from randomized clinical trials or recommendations from the American College of Obstetricians and Gynecologists or the Royal College of Obstetricians and Gynaecologists on standard of care for evaluation of postcoital bleeding [12].

## 2. Etiology

The differential diagnosis for women who present with postcoital bleeding is broad. Most women with postcoital bleeding have benign disease, which is reassuring given that the initial concern for both patient and provider is the possibility of underlying malignancy.  Table 1 outlines some of the most common causes for postcoital bleeding.

### 2.1. Cancer

The greatest fear for patients experiencing postcoital bleeding and providers taking care of these patients is the concern for underlying malignancy. Postcoital bleeding is the presenting complaint in 11% of women with cervical cancer [13]. Cervical cancer is the second most common cancer in women throughout the world. Annual global estimates for the year 2000 were 233,400 deaths and 470,600 new cases; in the United States in 2009, there were estimates that there were 11,270 new cases of cervical cancers and 4,070 deaths [17, 18]. The mean age for cervical cancer is 51.4 years [17]. The most important risk factor for this disease include women who have been infected with a high risk strain of the human papilloma virus (HPV), the virus believed to cause cervical cancer. Other risk factors include immunosuppression and smoking.  Table 2 illustrates the risk of cervical cancer in women with postcoital bleeding based on age [19]. The incidence of women with postcoital bleeding from cervical cancer has significantly decreased over the past decades due to enhanced screening for cervical cancer. Cervical cancer screening, via cervical cytology either with or without testing for HPV, allows for the identification of premalignant and malignant cervical disease, which is important given that CIN is largely asymptomatic [9, 20]. The most common histopathologic types of cervical cancer include squamous cell carcinoma (69%) and adenocarcinoma (25%) [21]. Of the two types, adenocarcinoma may be less likely to present with postcoital bleeding as lesions may be higher in the cervical canal and protected from the trauma of intercourse [1, 9]. Women presenting with postcoital bleeding who are found to have cervical cancer often are diagnosed with a higher stage of cancer than asymptomatic women [11, 22].

Although cervical cancer may be the initial concern of patients presenting with postcoital bleeding, vaginal cancer is another gynecologic malignancy for which postcoital bleeding may be the presenting symptom. Primary vaginal cancer is responsible for 3% of malignant neoplasms of the female genital tract. There are approximately 3000 cases diagnosed each year in the United States and approximately 900 deaths [23]. Vaginal intraepithelial neoplasia (VAIN), the precursor lesion to invasive vaginal carcinoma, is also rare with an incidence of approximately 0.2-0.3 cases per 100,000 women in the United States [24]. Most patients with VAIN or vaginal cancer are asymptomatic, but many women report postcoital spotting and unusual vaginal discharge [25]. Primary vaginal carcinoma can often be located on the posterior aspect of the upper one-third of the vagina. This area of the vagina has close proximity to the cervix in which it is believed that one of the most important risk factors for development of VAIN is from previous or concomitant cervical dysplasia [26, 27].

Cancer of the endometrium is the most common gynecologic cancer in the United States. In 2008, there were 40,100 cases of cancer of the endometrium and 7474 deaths attributed to this disease [28]. Vaginal bleeding in postmenopausal women is primarily secondary to atrophic changes, but this symptom can be the presenting complaint in 90% of women with endometrial carcinoma [29].

Finally, there are primary malignancies that may manifest in the lower genital tract and present with postcoital bleeding. Primary malignant lymphoma of the female genital tract is rare [30]. Non-Hodgkin's lymphoma has been found to be present in the cervix, vagina, and uterus. There are over one hundred reports of primary cervical non-Hodgkin's lymphoma of the cervix in which primary cervical lymphoma accounts for less than 1% of extranodal lymphomas [31]. Nevertheless, it is more common to have cervical involvement of lymphoma secondary to widespread disease [32].

### 2.2. Cervicitis

Cervicitis refers to an inflammation of the cervical stroma which can be either acute or chronic. Cervicitis typically presents with watery and mucopurulent discharge; however, postcoital bleeding is also associated with this condition. Acute cervicitis may be caused by infection with* C. trachomatis, N. gonorrhea, T. vaginalis, G. vaginalis*, and mycoplasma species [2]. Chronic cervicitis usually does not have an infectious source. Cervical infection is important to diagnose and treat early as this infection can ascend into the upper genital tract and lead to significant complications to include pelvic inflammatory disease, infertility, chronic pelvic pain, and increased risk for ectopic pregnancy.

### 2.3. Endometritis

Endometritis is an inflammation of the endometrium which can be either acute or chronic; differentiation is based on pathologic evaluation. Acute endometritis has the presence of microabscesses within the endometrial glands, whereas chronic endometritis has multiple plasma cells within the endometrial stroma [33, 34]. Chronic endometritis is often caused by infectious agents but can also be caused from foreign bodies, polyps, or fibroids within the uterine cavity; nevertheless, no identifiable source is found in one-third of patients [35]. Most women with symptomatic chronic endometritis can present with heavy menstrual bleeding or intermenstrual bleeding; however, some women may initially complain of postcoital bleeding.

### 2.4. Cervical Polyps

Cervical polyps are not an infrequent incidental finding during speculum exams and can be a source of postcoital bleeding secondary to cervical trauma with intercourse. Both endocervical and cervical polyps are the most common benign neoplastic growth that occurs on the cervix with an incidence of 4% of gynecologic patients [36]. Polyps typically occur in multiparous patients in their 40s to 50s. Most patients with cervical polyps only have one, but it is not uncommon to have more than one. On gross examination, they appear as smooth, reddish purple lobular structures that are friable and bleed easily when touched. Most polyps are only a few centimeters in size. Polyps may arise from the endocervical portion of the cervix or appear on the cervical portio. It is believed that these polyps originate from recurrent inflammation of the cervix versus focal response to hormonal stimulation.

### 2.5. Cervical Ectropion

Cervical ectropion refers to the eversion of the endocervix which exposes the columnar epithelium to the vaginal milieu. It is important to note that the presence of ectropion does not indicate a pathologic condition. This area of the cervix may have a reddish appearance and be covered with yellow discharge in which most women with symptomatic cervical ectropion complain of vaginal discharge. This condition is often seen during adolescence, women taking oral contraceptive pills, and pregnancy due to the remodeling process of the cervix. The exposure of the columnar epithelium of the endocervix to the vagina then increases the risk of bleeding with intercourse due to the friability of these cells [37].

### 2.6. Pelvic Organ Prolapse

Pelvic organ prolapse refers to the herniation of pelvic organs [cervix, bladder, rectum, and uterus] to or beyond the vaginal walls. It is hard to determine the exact prevalence of pelvic organ prolapse for multiple reasons: most women only present when symptoms become severe, providers are poor at screening women during routine visits, many women are embarrassed to report these symptoms to providers, and women with minor prolapse often do not report these symptoms to their providers. Risk factors for pelvic organ prolapse include parity, obesity, age, hysterectomy, race, constipation, and chronic cough. There can be significant irritation and trauma to the vagina and cervix when these organs prolapse through the introitus which can lead to postcoital bleeding [38].

### 2.7. Vaginal/Vulvar Etiologies

Vaginal atrophy, also known as urogenital atrophy, atrophic vaginitis, or vulvovaginal atrophy, results from a loss of estrogen which can lead to vulvovaginal complaints such as postcoital bleeding. This condition typically occurs in menopausal women but may also occur in women who experience a decrease in estrogen. Other complaints include vaginal dryness, vaginal burning, dyspareunia, decreased lubrication, vaginal discharge, and pelvic pressure. Lastly, lichenoid lesions such as lichen planus and lichen sclerosis may also lead to postcoital bleeding.

### 2.8. Benign Vascular Neoplasms

Vascular tumors of the female genital tract are rare [39]. These lesions include hemangiomas, lymphangiomas, angiomatosis, and arteriovenous malformation. Most tumors are found incidentally on exam due to their asymptomatic nature. However, when symptomatic, postcoital bleeding may be a symptom associated with these conditions [40].

### 2.9. Sexual Abuse

Domestic and sexual abuse is a serious public health problem in the United States by which 32 million Americans are affected [41]. Gynecologists should screen women for abuse at every single visit regardless of complaints. For example, one study demonstrated that 5.6% of women were diagnosed with sexual abuse prior to instituting a universal screening program, whereas, after implementation of universal screening, 30% of the population was found to be affected by abuse [42]. Depending on the extent of the abuse, victims may experience significant genital trauma.

## 3. Diagnosis

**Figure 1:**
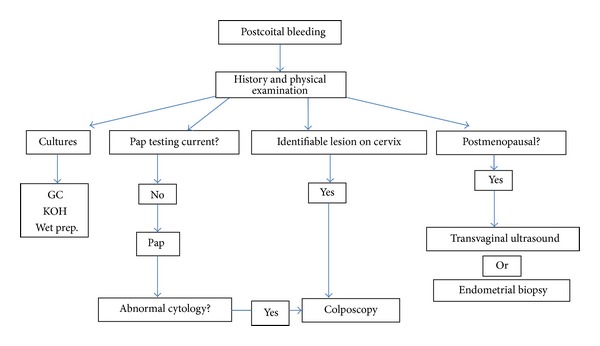
Diagnostic approach to postcoital bleeding.

At this time, there are no established guidelines from the American College of Obstetricians and Gynecologists or the Royal College of Obstetricians and Gynaecologists or evidence from randomized clinical trials to base recommendations on diagnosis and treatment of postcoital bleeding. The following discussion provides various considerations to take into account when approaching a patient with postcoital bleeding.  Figure 1 presents a diagnostic algorithm for women with postcoital bleeding.

### 3.1. History

A thorough emphasis on patient history often leads to an accurate diagnosis of postcoital bleeding. With all gynecologic patients, it is important to obtain an accurate menstrual history. Factors which should be elicited from the patient include the frequency of the patient's menstrual cycle, days of menstruation, presence of heavy bleeding, presence of intermenstrual bleeding, and whether cycles are regular or irregular. The duration of normal menstrual flow is 5 days with cycles typically lasting between 21–35 days [43]. Clinicians should also evaluate if the patient is postmenopausal which is defined as 12 months of amenorrhea without any other physiologic or pathologic cause. Moreover, history should focus on whether the patient's postcoital bleeding is truly bleeding that occurs as a direct result of intercourse or if it is secondary to irregular menstrual bleeding. History may also help to differentiate between whether bleeding is originating from the uterus or cervix. Patients with abnormal uterine bleeding often report heavy periods, intermenstrual bleeding not related to intercourse, and irregular menstrual cycles.

There are multiple considerations to take into account for patients past medical history. Screening should be performed as to whether the patient has been diagnosed or has any symptoms concerning a bleeding disorder. Regarding surgical history, determine whether there have been surgeries on the genital tract with focus on timing and indication for the surgery. A detailed sexual history should be obtained with focus on number of partners, new partners, and history of any sexually transmitted infections for either the patient or her partners. It is imperative to also screen patients for domestic abuse and/or sexual abuse as genital tract trauma can lead to postcoital bleeding. Patients may not be willing to volunteer this information for either embarrassment or fear of retaliation. Providers should attempt to establish rapport with the patient and create an environment in which patients may be willing to share this information. If the patient's partner is present, then strategies may be employed to have the partner step outside the exam room during the time of pelvic exam, at which point one may also evaluate the patient privately for concerns of abuse. Finally, providers should ensure cervical cancer screening is up-to-date.

There are also multiple factors to ask on review of symptoms that can help establish a diagnosis. For example, one should inquire about pain with focus on pain during menstruation (dysmenorrhea) or with intercourse (dyspareunia). Regarding the latter, a detailed history should be obtained as to when the dyspareunia occurs: at all times, with deep penetration, or in certain positions. Patients should be asked if there has been any change in discharge, specifically color, consistency, frequency, and odor. Finally, patients should be screened for symptoms concerning for pelvic organ prolapse such as a feeling of heaviness in the vagina, sensation that things are dropping, need to splint in order to have bowel movement or urination, and visualization of organs prolapsing from the vagina.

### 3.2. Physical Examination

Every woman presenting with postcoital bleeding requires a thorough examination of the genital tract. A bivalve speculum exam should be performed to evaluate the vaginal rugae and cervix. Attention should be focused to determine if there are any lacerations or trauma to the vaginal walls. Upon examining the cervix, one should evaluate any obvious gross lesions on the cervix or lesions protruding through the cervical canal. Colposcopy may be considered if there are any suspicious lesions on the cervix to further evaluate the lesion under high power. In obtaining cultures or clearing mucus from the cervix, one should also determine whether gentle palpation alone of the cervix with a swab is able to recreate bleeding.

Considerations may then be made to break down the bivalve speculum and perform an inspection of the vagina with one blade of the speculum. This may allow for a better visualization of the vaginal rugae as there is less risk of obstruction by the blades of the speculum. This technique may be used to evaluate signs of pelvic organ prolapse. A blade should be placed along the anterior vaginal wall, while having the patient Valsalva, to evaluate prolapse of the posterior structures.

A bimanual exam is performed to evaluate the size and contour of the uterus as well as the presence of any adnexal masses. During this exam, one may delineate whether there is presence of cervical motion tenderness which may help with diagnosing an underlying infection. If the patient has complained of dyspareunia or pelvic pain, then it is also important to delineate the location of the pain. Most women will not find a bimanual exam comfortable, so it is important to specifically ask what on exam reproduces the patient's pain. Finally, if there is concern for underlying malignancy, then one should also evaluate the inguinal lymph nodes to determine if there is any lymphadenopathy. A rectovaginal exam should be performed to determine if there are any masses or nodularities located on the anterior surface of the rectum or extension of disease into the parametrium.

### 3.3. Laboratory Tests

On speculum exam, there are multiple cultures that may be obtained to further evaluate postcoital bleeding. Nucleic acid amplification testing (NAAT) for* N. gonorrhoeae*,* C. trachomatis, *and* T. vaginalis* should routinely be obtained in women presenting with postcoital bleeding. Even though wet mount is the most cost-effective means of diagnosing* Trichomonas*, the overall sensitivity is low and is dependent on the inoculum size; thus, NAAT testing has become popular due to its relatively high sensitivity and specificity. Women who are not recent on cervical cancer screening may also undergo cervical cytology, with or without testing for high risk HPV. Nevertheless, it is important to note that the false negative rate for Pap smears in the presence of invasive cancer is 50%; thus, gynecologists must be cognizant that a normal smear does not rule out underlying malignancy in women presenting with postcoital bleeding [44].

There are multiple variations based on expert opinion on which patients with postcoital bleeding should be referred for colposcopy. There is little debate that women with an abnormal pap smear or a grossly visible lesion that is suspicious for an underlying malignancy should be referred for colposcopy. Nevertheless, there is controversy on whether colposcopy should be performed on women with no visible lesions and negative cervical cancer screening results on recently performed testing. One may argue that postcoital bleeding alone is not an absolute indication for colposcopy [12]. Providers should discuss with their patients that there are no guidelines or evidence to base recommendations in these scenarios [19]. One retrospective study of 314 women with postcoital bleeding seen by a gynecologic service found that 20% of women diagnosed with cervical cancer or vaginal cancer on colposcopy had a normal speculum exam with negative cytology prior to the procedure [11]. In short, there is limited evidence to base recommendations on colposcopy for women with negative Pap smears and no obvious lesion on exam. However, the Working Group of the Royal Australian College of General Practice and of Obstetrics and Gynecology [7] recommend that general practitioners refer women for colposcopy if they have one of the following qualifications; nevertheless, it is important to realize that these recommendations are not evidence based [15]:

1. persistent postcoital bleeding,
2. postcoital bleeding associated with a single smear suggestive of LGSIL or worse,
3. postcoital bleeding associated with repeated smears with minor atypia or wart virus changes.

Directed biopsy with colposcopy remains the standard for disease detection [43]. Recent studies, however, have compared directed biopsy to blind four-quadrant ectocervical biopsies or loop excision procedure as diagnostic criteria [45, 46]. These studies found that the presence of CIN 2 and higher was missed on directed biopsy but detected on random four-quadrant biopsies in 18.6–31.6% of times [46, 47]. Another study, however, demonstrated that diagnosis of CIN 2 and higher was found in 57.1% of women with colposcopy directed biopsy versus 37.4% with random biopsy [48]. Based on these studies, the American College of Obstetricians Gynecologists recommends that biopsies should be performed on all visible lesions [49]. These recommendations and studies pertain to patients with abnormal cytology. It is hard to interpret these recommendations in women with postcoital bleeding and no history of abnormal cytology.

There are multiple ways to evaluate the endocervical and endometrial cavity for sources of postcoital bleeding. One option is to perform an office endometrial biopsy which can evaluate for the presence of endometrial hyperplasia, malignancy, and endometrial polyps. If the patient is not amenable to this procedure or if further imaging is indicated, then a saline infused sonohysterogram is another useful diagnostic technique to evaluate the contours of the uterine cavity. Finally, depending on the presence of other complaints, one may also consider diagnostic hysteroscopy to evaluate the cervical canal and uterine cavity; although this procedure should be reserved for patients with complaints of abnormal uterine bleeding which may suggest an endometrial source for the abnormal bleeding.

The clinical approach to postmenopausal women presenting with postcoital bleeding warrants other considerations to exclude carcinoma of the endometrium. The American College of Obstetricians and Gynecologists reports that there are two acceptable methods for evaluating malignancy: endometrial biopsy or transvaginal ultrasonography. An endometrial thickness of greater than 4 mm in a patient with postmenopausal bleeding requires further evaluation with sonohysterography, office endometrial biopsy, or hysteroscopy. Alternatively, providers may also decide to initiate the evaluation of postmenopausal bleeding with performing an endometrial biopsy [50].

## 4. Management

The majority of women presenting to their primary care physician with the complaints of postcoital bleeding will be found to have no obvious underlying cause for their bleeding based on history, exam, or laboratory investigation [11]. Nevertheless, the reassuring aspect is that 60% of naturally menstruating women with postcoital bleeding will have spontaneous resolution of symptoms within six months [4]. Half of these women will maintain resolution for two years [4].

### 4.1. Infection

Any woman who is found to have evidence of genital tract infection should be immediately treated to prevent long term repercussions. Treatment options should be guided based on laboratory and microscopy findings. With respect to a clinical diagnosis of pelvic inflammatory disease, treatment should not be withheld if testing for chlamydia and gonorrhea are negative as the three major criteria needed for the diagnosis of pelvic inflammatory disease per the Centers for Diseases Control and the World Health Organization include cervical motion tenderness, bilateral adnexal tenderness, and abdominal tenderness.

### 4.2. Cervical Ectropion

Cervical ectropion does not require treatment unless bleeding is persistent and bothersome to the patient. Prior to proceeding with treatment, one should ensure that they have ruled out underlying malignancy as certain treatments for cervical ectropion may mask or exacerbate malignant lesions. Cervical ablation with either cryotherapy or electrocautery is effective in mitigating further postcoital bleeding. However, there are significant side effects to include copious vaginal discharge until healing is complete and cervical stenosis which can affect subsequent pregnancies [51]. An alternative therapy may be to use acidifying agents such as boric acid suppositories 600 mg vaginally at bedtime [52].

### 4.3. Polyps

Clinicians should consider removal of symptomatic polyps or when they appear atypical with concerns for malignancy. A cervical polypectomy can often be performed in the office without sedation. Removal is performed by first placing a speculum into the vagina to visualize the cervical polyp. A forcep may then be used to grasp the polyp at its base and twist it off. If the base is visualized, then cauterization should be performed to prevent further bleeding. All polyps that are removed should be sent to pathology to be evaluated for malignancy [52–54]. Furthermore, if there is concern for endometrial polyps, then the patient should be referred to operative hysteroscopy with possible dilation and curettage.

### 4.4. Cancer

Colposcopy with directed biopsies is indicated for patients with abnormal cytology. If patients are found to have CIN on cervical biopsy, then one may follow the guidelines established by the American College of Obstetricians and Gynecologists or the American Society for Colposcopy and Cervical Pathology to determine whether the patient needs to be referred for an excisional procedure versus surveillance. Patients who are found to have genital tract cancer such as vaginal or cervical cancer should be referred to a gynecologic oncologist for further evaluation and treatment.

### 4.5. Vaginal Atrophy

Postcoital bleeding associated with vaginal dryness may first be treated with vaginal moisturizers and lubricants which can be used prior to and during intercourse. Although these methods may assist with ameliorating discomfort during intercourse, they do not have any direct effect on improving atrophic changes. Women who continue to experience postcoital bleeding despite lubricants may require vaginal estrogen therapy. Estrogen therapy is one of the most effective treatment options for vaginal atrophy as it thickens the vaginal epithelium and decreases dryness. Low dose vaginal estrogen therapy should be the first line treatment for postmenopausal women with only vaginal complaints as it is more effective and also prevents possible side effects of systemic treatment. Special considerations should be made with use of estrogen therapy for women who have breast cancer and/or cardiovascular disease.

## 5. Conclusion

Postcoital bleeding can be an annoying complaint for patients and a worrisome symptom for providers due to the risk of underlying malignancy. Despite being a common gynecologic problem, there is large diversity among gynecologists on the management of postcoital bleeding [55]. Unlike abnormal uterine bleeding or the management of abnormal cytology, there are no recommendations from governing bodies on the management of postcoital bleeding. Patients presenting with postcoital bleeding require a full history and physical examination to help in developing a differential diagnosis to guide evaluation and treatment. Although most patients with postcoital bleeding do not have underlying malignancy, providers must pay close attetion to ensure that appropriate screening tests are up-to-date. Physicians should also be aware that a large portion of women presenting with postcoital bleeding will not have an obvious source for their bleeding; however, as long as malignancy is ruled out, most of these women's symptoms will naturally resolve in premenopausal women.

## Figures and Tables

**Table 1:** Common causes of postcoital bleeding.

Benign growths	
Endometrial polyps	
Cervical polyps	
Cervical ectropion	
Infection	
Cervicitis	
Pelvic inflammatory disease	
Endometritis	
Vaginitis	
Genital/vulvar lesions	
Herpes simplex virus	
Syphilis	
Chancroid	
Lymphogranuloma venereum	
Condyloma accuminata	
Benign conditions	
Vaginal atrophy	
Pelvic organ prolapse	
Benign vascular neoplasms	
Endometriosis	
Malignancy	
Cervical cancer	
Vaginal cancer	
Endometrial cancer	
Trauma	
Sexual abuse	
Foreign bodies	

**Table 2:** Risk of cervical cancer in women with postcoital bleeding.

Age (years)	Risk
20–24	1 : 44,000
25–34	1 : 5,600
35–44	1 : 2,800
45–54	1 : 2,400

## References

[1] Viikki M, Pukkala E, Hakama M (1998). Bleeding symptoms and subsequent risk of gynecological and other cancers. *Acta Obstetricia et Gynecologica Scandinavica*.

[2] Lindner LE, Geerling S, Nettum JA, Miller SL, Altman KH (1988). Clinical characteristics of women with chlamydial cervicitis. *Journal of Reproductive Medicine for the Obstetrician and Gynecologist*.

[3] Shapley M, Jordan K, Croft PR (2004). An epidemiological survey of symptoms of menstrual loss in the community. *British Journal of General Practice*.

[4] Shapley M, Blagojevic-Bucknall M, Jordan K, Croft P (2013). The epidemiology of self-reported intermenstrual and postcoital bleeding in the perimenopausal years. *British Journal of Obstetrics and Gynaecology*.

[5] Tehranian A, Rezaii N, Mohit M, Eslami B, Arab M, Asgari Z (2009). Evaluation of women presenting with postcoital bleeding by cytology and colposcopy. *International Journal of Gynecology and Obstetrics*.

[6] Selo-Ojeme DO, Dayoub N, Patel A, Metha M (2004). A clinico-pathological study of postcoital bleeding. *Archives of Gynecology and Obstetrics*.

[7] Fraser IS, Petrucco OM (1996). Management of intermenstrual and postcoital bleeding, and an appreciation of the issues arising out of the recent case of O’Shea versus Sullivan and Macquarie pathology. *Australian and New Zealand Journal of Obstetrics and Gynaecology*.

[8] Pardanani NS, Tischler LP, Brown WH, de Feo E (1975). Carcinoma of cervix. evaluation of treatment in community hospital. *New York State Journal of Medicine*.

[9] Pretorius R, Semrad N, Watring W, Fotheringham N (1991). Presentation of cervical cancer. *Gynecologic Oncology*.

[10] Shalini R, Amita S, Neera MA (1998). How alarming is post-coital bleeding—a cytologic, colposcopic and histopathologic evaluation. *Gynecologic and Obstetric Investigation*.

[11] Rosenthal AN, Panoskaltsis T, Smith T, Soutter WP (2001). The frequency of significant pathology in women attending a general gynaecological service for postcoital bleeding. *British Journal of Obstetrics and Gynaecology*.

[12] Jha S, Sabharwal S (2002). Outcome of colposcopy in women presenting with postcoital bleeding and negative or no cytology—results of a 1-year audit. *Journal of Obstetrics and Gynaecology*.

[13] Anorlu RI, Abdul-Kareem FB, Abudu OO, Oyekan TO (2003). Cervical cytology in an urban population in Lagos, Nigeria. *Journal of Obstetrics and Gynaecology*.

[14] Department of Health Referral guidelines for suspected cancer. http://www.dh.gov.uk/en/Publicationsandstatistics/Publications/PublicationsPolicyAndGuidance/DH_4008746.

[15] Khattab AF, Ewies AA, Appleby D, Cruickshank DJ (2005). The outcome of referral with postcoital bleeding (PCB). *Journal of Obstetrics and Gynaecology*.

[16] National Health Service Cervical Screening Programme (NHSCP) Colposcopy and programme management guidelines for the NHS cervical screening programme. http://www.cancerscreening.nhs.uk/cervical/publications/nhscp20.pdf.

[17] Parkin DM, Bray F, Ferlay J, Pisani P (2001). Estimating the world cancer burden: globocan 2000. *International Journal of Cancer*.

[18] Jemal A, Siegel R, Ward E (2009). Cancer statistics, 2009. *CA: A Cancer Journal for Clinicians*.

[19] Shapley M, Jordan J, Croft PR (2006). A systematic review of postcoital bleeding and risk of cervical cancer. *British Journal of General Practice*.

[20] Khan Z, Appleton F, Turner J (2008). Is cervical intra-epithelial neoplasia symptomatic?. *Journal of Obstetrics and Gynaecology*.

[21] Kurman RJ, Norris HJ, Wilkinson EJ (1992). *Atlas of Tumor Pathology: Tumors of the Cervix, Vagina, and Vulva*.

[22] de Souza NM, Soutter WP, McIndoe GA, Gilderdale DJ, Krausz T (1997). Stage I cervical cancer: tumor volume by magnetic resonance imaging of screen-detected versus symptomatic lesions. *Journal of the National Cancer Institute*.

[23] Siegel R, Ma J, Zou Z, Jemal A (2014). Cancer statistics, 2014. *CA: A Cancer Journal for Clinicians*.

[24] Henson D, Tarone R (1977). An epidemiologic study of cancer of the cervix, vagina, and vulva based on the Third National Cancer Survey in the United States. *The American Journal of Obstetrics and Gynecology*.

[25] Zelings KP, Byrd K, Tarney CM (2013). A clinicopathologic study of vaginal intraepithelial neoplasia. *Obstetrics & Gynecology*.

[26] Choo YC, Anderson DG (1982). Neoplasms of the vagina following cervical carcinoma. *Gynecologic Oncology*.

[27] Geelhoed GW, Henson DE, Taylor PT, Ketcham AS (1976). Carcinoma in situ of the vagina following treatment for carcinoma of the cervix: a distinctive clinical entity. *The American Journal of Obstetrics and Gynecology*.

[28] American Cancer Society Cancer facts and figures 2008. http://www.cander.org/downloads/STT/2008CAFFfinalsecured.pdf.

[29] Goldstein RB, Bree RL, Benson CB (2001). Evaluation of the woman with postmenopausal bleeding: society of radiologists in ultrasound-sponsored consensus conference statement. *Journal of Ultrasound in Medicine*.

[30] Abu Amna F, Howell R, Raj S (2009). Lymphoma of the cervix uteri. *BMJ Case Reports*.

[31] Hanley KZ, Tadros TS, Briones AJ, Birdsong GG, Mosunjac MB (2009). Hematologic malignancies of the female genital tract diagnosed on liquid-based pap test: cytomorphologic features and review of differential diagnoses. *Diagnostic Cytopathology*.

[32] Grace A, O’Connell N, Byrne P (1999). Malignant lymphoma of the cervix: an unusual presentation and a rare disease. *European Journal of Gynaecological Oncology*.

[33] Kurman R, Mazur M, Blaustein A (1982). Benign disease of the endometrium. *Pathology of the Female Genital Tract*.

[34] Greenwood SM, Moran JJ (1981). Chronic endometritis: morphologic and clinical observations. *Obstetrics and Gynecology*.

[35] Vasudeva K, Thrasher TV, Richart RM (1972). Chronic endometritis: a clinical and electron microscopic study. *The American Journal of Obstetrics and Gynecology*.

[36] Farrar HK, Nedoss BR (1961). Benign tumors of the uterine cervix. *The American Journal of Obstetrics and Gynecology*.

[37] Goldacre MJ, Loudon N, Watt B (1978). Epidemiology and clinical significance of cervical erosion in women attending a family planning clinic. *British Medical Journal*.

[38] Mawajdeh SM, Al-Qutob RJ, Farag AM (2003). Prevalence and risk factors of genital prolapse: a multicenter study. *Saudi Medical Journal*.

[39] Pethe VV, Chitale SV, Godbole RN, Bidaye SV (1991). Hemangioma of the ovary—a case report and review of literature. *Indian Journal of Pathology and Microbiology*.

[40] Gerbie AB, Hirsch MR, Green RR (1955). Vascular tumours of female genital tract. *Obstetrics & Gynecology*.

[41] Tjaden P, Thoennes N (2000). *Extent, Nature, and Consequences of Intimate Partner Violence: Findings from the National Violence against Women Survey*.

[42] McLeer SV, Anwar R (1989). A study of battered women presenting in an emergency department. *The American Journal of Public Health*.

[43] Committee on Practice Bulletins-Gynecology (2012). Practice bulletin no. 128: diagnosis of abnormal uterine bleeding in reproductive-aged women. *Obstetrics & Gynecology*.

[44] Sasieni PD, Cuzick J, Lynch-Farmery E (1996). Estimating the efficacy of screening by auditing smear histories of women with and without cervical cancer. The National Co-ordinating Network for Cervical Screening Working Group. *British Journal of Cancer*.

[45] Solomon D, Davey D, Kurman R (2002). The 2001 Bethesda system: terminology for reporting results of cervical cytology. *Journal of the American Medical Association*.

[46] Cuzick J, Szarewski A, Cubie H (2003). Management of women who test positive for high-risk types of human papillomavirus: the HART study. *The Lancet*.

[47] Kjaer SK, van den Brule AJC, Paull G (2002). Type specific persistence of high risk human papillomavirus (HPV) as indicator of high grade cervical squamous intraepithelial lesions in young women: population based prospective follow up study. *British Medical Journal*.

[48] Pretorius RG, Zhang W-H, Belinson JL (2004). Colposcopically directed biopsy, random cervical biopsy, and endocervical curettage in the diagnosis of cervical intraepithelial neoplasia II or worse. *The American Journal of Obstetrics and Gynecology*.

[49] American College of Obstetricians and Gynecologists (2008). ACOG practice bulletin No. 99: management of abnormal cervical cytology and histology. *Obstetrics & Gynecology*.

[50] ACOG Committee Opinion No. 440 (2009). The role of transvaginal ultrasonography in the evaluation of postmenopausal bleeding. *Obstetrics & Gynecology*.

[51] Kong GWS, Yim SF, Cheung TH, Chung TKH (2009). Cryotherapy as the treatment modality of postcoital bleeding: a randomised clinical trial of efficacy and safety. *Australian and New Zealand Journal of Obstetrics and Gynaecology*.

[52] Belfiore P, Costa E, de Cantis S, Vassallo R, Marino A (2005). Effectiveness and persistence of a topical treatment for cervical ectropion with deoxyribonucleic acid. *Minerva Ginecologica*.

[53] Kerner H, Lichtig C (1993). Mullerian adenosarcoma presenting as cervical polyps: a report of seven cases and review of the literature. *Obstetrics and Gynecology*.

[54] van Renterghem N, de Paepe P, van den Broecke R, Bourgain C, Serreyn R (2005). Primary lymphoma of the cervix uteri: a diagnostic challenge: report of two cases and review of the literature. *European Journal of Gynaecological Oncology*.

[55] Alfhaily F, Ewies A (2009). Postcoital bleeding: a study of the current practice amongst consultants in the United Kingdom. *European Journal of Obstetrics Gynecology and Reproductive Biology*.

